# Social media as an emerging tool for reducing prescription opioid misuse risk factors

**DOI:** 10.1016/j.heliyon.2020.e03471

**Published:** 2020-03-06

**Authors:** Sean D. Young, Sung-Jae Lee, Hendry Perez, Navkiran Gill, Lillian Gelberg, Keith Heinzerling

**Affiliations:** a Department of Informatics, School of Information and Computer Sciences, University of California, Irvine, CA, USA; b Department of Emergency Medicine, School of Medicine, University of California, Irvine, CA, USA; c Department of Psychiatry and Biobehavioral Sciences, David Geffen School of Medicine, University of California, Los Angeles, CA, USA; d Department of Family Medicine, David Geffen School of Medicine, University of California, Los Angeles, CA, USA; e University of California Institute for Prediction Technology, David Geffen School of Medicine, University of California, Los Angeles, CA, USA

**Keywords:** Public health, Information science, Information technology, Social media, Applied psychology, Chronic pain, Opioid crisis, SOCIAL MEDIA, Opioids, Addiction

## Abstract

Interventions are urgently needed to reduce prescription opioid misuse risk factors, including anxiety and concomitant use of sedatives. However, only a limited number of randomized controlled opioid intervention trials have been conducted. We sought to determine whether an online behavior change/support community, compared to a control Facebook group, could reduce anxiety and opioid misuse among chronic pain patients. 51 high-risk non-cancer chronic pain patients were randomly assigned to either a Harnessing Online Peer Education (HOPE) peer-led online behavior change intervention or a control group (no peer leaders) on Facebook for 12 weeks. Inclusion criteria were: 18 years or older, a UCLA Health System patient, prescribed an opioid for non-cancer chronic pain between 3 and 12 months ago, and a score of ≥9 on the Current Opioid Misuse Measure (COMM) and/or concomitant use of benzodiazepines. Participation in the online community was voluntary. Patients completed baseline and follow-up assessments on Generalized Anxiety Disorder screener (GAD-7), COMM, and frequency of social media discussions about pain and opioid use. Compared to control group participants, intervention participants showed a baseline-to-follow-up decrease in anxiety, and more frequently used social media to discuss pain, prescription opioid use, coping strategies, places to seek help, and alternative therapies for pain. Both groups showed a baseline to follow-up decrease in COMM score. Preliminary results support the use an online community interventions as a low-cost tool to decrease risk for prescription opioid misuse and its complications.

## 1 Introduction

Prescription opioid misuse and abuse have reached epidemic proportions, nearly quadrupling from 2000 to 2014 [1]. A 2013 study found that prescription opioids were the most frequently misused of all prescription medications, and accounted for more fatal overdoses than cocaine and heroin combined [2, 3]. Despite these complications, opioid prescribing for chronic pain skyrocketed during those years [4]. In 2012, enough prescription pain killers were prescribed (259 million) for every American adult to have a bottle of pills [5].

Interventions to reduce risk factors for opioid misuse and abuse, including anxiety and concomitant use of sedatives, are urgently needed. Interventions that successfully reduce anxiety and risk for prescription opioid misuse might have a particularly important public health impact due to the large number of chronic opioid patients with comorbid mood disorders [6, 7]. Anxiety is a significant risk factor for opioid misuse and abuse, however; a limited number of randomized controlled opioid intervention trials have been conducted to date, with no studies showing improvements in anxiety [8].

Social media and online communities, such as Facebook groups, may serve as low-cost, innovative platforms for delivering wide-spread community-based social support interventions among chronic opioid patients. Online behavior change platforms have already been used in a variety of public health and medical studies. For example, participants in the Harnessing Online Peer Education (HOPE) HIV intervention, a peer-led online community designed to stimulate HIV testing among at-risk populations, were more than twice as likely to change their behavior and get an HIV test compared to a control group [9]. The HOPE intervention integrates social normative and diffusion of innovations theories into an online platform and has been particularly effective in increasing communication and behavior change for stigmatizing behaviors, such as substance use [9, 10]. HOPE online community interventions are based on psychological theory and approaches on how to reduce stigma [11, 12, 13, 22], and are therefore applicable to chronic pain and opioid patients because of the stigmatization associated with these populations. Patients taking prescription opioids have already suggested integrating online support communities such as HOPE into their treatment [14], however, no known studies have explored whether and how these technologies might be applied.

In this pilot study, we sought to explore the feasibility and preliminary efficacy of using a HOPE Facebook community, compared to a control Facebook community, with a specific focus on whether the intervention translates to reduced anxiety and opioid misuse among non-cancer pain patients prescribed opioids. A detailed description on how the HOPE intervention was adapted for this population is published elsewhere [15].

## 2 Materials and methods

Patients had to meet a two-step screening process for study inclusion: 1) verified as being a current UCLA health system patient on chronic opioid therapy, and 2) diagnosed as high-risk for opioid misuse and willing to join an online support group (further details below). Feasibility was assessed based on participants accepting invites to join the online community. Preliminary efficacy was assessed based on tests for preliminary differences in primary outcomes (below) resulting from the intervention.

Step 1 Upon registering with the UCLA health system at intake, patients completed a number of standard forms and questionnaires, including allowing researchers to contact them for potential studies. Based on these patient data, we received contact information for 5,358 patients who had been identified through the UCLA medical records registry, xDR, and satisfied the following inclusion filtering criteria: 18 years of age or older, prescribed an opioid for non-cancer chronic pain between 3 and 12 months ago, and had listed a contact email and/or phone number. We emailed 2,665 (49.7%) of patients who provided email addresses in their contact information to invite them to screen for further eligibility by completing the Current Opioid Misuse Measure (COMM, 17 items) questionnaire as well as indicating whether they were concurrently taking benzodiazepines with their opioids. COMM is a brief 17-item assessment to monitor chronic pain patients on opioid therapy for aberrant medication-related behaviors and elevated risk of opioid misuse [16]. Each item is rated on a 5-point rating scale ranging from 0 (“never”) to 4 (“very often”), with a total COMM score being computed by summing the 17-items (range = 0-68). A score of 9 or higher is considered positive for risk of opioid misuse. 417 participants clicked on a link to automatically opt-out. We received complete responses on the COMM from 249 (9.3%) patients. 76 out of 249 (30.5%) patients were eligible to participate, based on receiving a score of 9 or greater (the cut-off point for opioid misuse) on the COMM [16] and/or self-reporting concomitant use of benzodiazepines. 63 out of 76 patients (82.9%) consented to participate.

Step 2 After informed consent, participants completed a self-administered baseline survey. Measures included the Generalized Anxiety Disorder screener (GAD-7), Brief Pain Inventory, Health-related Quality of Life (HRQoL), demographic characteristics, and use of social media to discuss pain and opioid-related issues. Anxiety was assessed using the GAD-7 score [17], which was developed for clinical applications and uses the clinical diagnostic criteria for generalized anxiety disorder from the Diagnostic and Statistical Manual of Mental Disorders [18]. Items reference feelings of anxiousness, worry, fear, and irritability occurring over the previous 2 weeks and are rated on a frequency scale from 0 (“not at all”) to 3 (“nearly every day”). Sum scores are typically reported (range: 0-21), with scores of 0–4, 5 to 9, 10 to 14, and 15 to 21 having been proposed to differentiate between minimal, mild, moderate, and severe symptom experiences [17]. 51 of the 63 (81.0%) consented patients completed the full baseline assessment, accepted a request to be added to a Facebook group, and were enrolled in the study.

Enrolled participants were randomly assigned using a random number generator to an intervention (n = 26) or control (n = 25), closed, secret group on Facebook for 12 weeks from July to October of 2016. Intervention group participants were assigned to a group moderated by peer role models who had attending training sessions on how to use online communities to encourage discussions around chronic pain and safe opioid use. Participants in the control group were assigned to a Facebook community group without peer role models. Participants in both groups were informed they could use the group to discuss any topic of interest, hopefully in a way that could reduce their pain and risk for opioid misuse. After participants were enrolled and joined the Facebook group, further participation in the online community was voluntary. Participants were paid in online gift cards to complete research assessments at baseline ($30) and 12-week follow-up ($40), including the COMM (which was administered at screening and follow-up).

Peer leaders in the intervention group were UCLA patients, 18 or older, reportedly taking an opioid prescribed for chronic pain, and recommended by a study physician as being sociable and effectively managing prescription opioids. Eight peer leaders were recruited and attended 2 in-person training sessions (4 h each) at UCLA on chronic pain and opioid epidemic-related epidemiology, and how to use social media to build communities discussing chronic pain issues and opioid-related safe behavior change. Peer leaders were instructed to not provide clinical recommendations, but to focus on stimulating conversations around pain and personal experiences. A number of peer leaders were either unable to attend both in-person training sessions or were unavailable after training, stating that their pain had worsened. Three of the peer leaders attended both training sessions and were available for the 12-week online intervention.

The study team worked with the peer leaders each week to guide them on topics to discuss with patients. For example, in the first 4 weeks, peer leaders were encouraged to attempt to build trust with other patients through friendly conversations. In later weeks, peer leaders were encouraged to share personal stories about their own chronic pain and opioid-related issues along with their attempts to overcome them. Each week, peer leaders were assigned to both directly message patients and create public wall posts on assigned topics, as well as to complete weekly tracking sheets detailing the process. This information was used to help guide the following week's recommendations to peer leaders on how to improve online community engagement. Peer leaders were paid in online gift cards for completing these tracking sheets every week ($30 for the first 4 weeks, $40 for the second four weeks, and $50 for the last four weeks). Sample size for this pilot was determined based on assessing group differences in social media-based conversations related to chronic pain and opioid use.

Chi-square and t-tests were used to assess baseline demographic differences between the intervention and control groups. Chi-square tests were used to compare the social media communications by intervention and the control group at baseline and follow-up. Paired t-tests were used to compare COMM and GAD-7 scores pre- (baseline) and post-intervention (12 weeks). Analyses were conducted using SAS software, version 9.4. The assumption of normality was tested using the chi-square test for normality. The UCLA Institutional Review Board (IRB) approved this study (ClinicalTrials.gov: NCT02735785).

## 3 Results

Six (6) intervention group patients and 7 control group patients did not complete follow-up assessments, including one deceased patient, leaving 38 participants (75%) with complete baseline and follow-up assessments. There were no statistically significant differences between groups that completed both baseline and follow-up assessments, compared to those who only finished the baseline assessment. Among those who completed baseline and follow-up assessments, there were no statistically significant baseline differences between groups on any variables (Table 1).

**Table 1:** Demographic characteristics of patients, Los Angeles, CA, USA.

	Intervention Group (n = 20)	Control Group (n = 18)	Total (n = 38)	P Value^a^
Age, Mean (SD)	45.8 (14.0)	45.8 (15.9)	45.8 (14.7)	.99

Gender				

Male	6 (30%)	7 (39%)	13 (34%)	
Female	14 (70%)	11 (61%)	25 (66%)	.56

Race/Ethnicity				

White/European Decent	11 (40%)	12 (77%)	23 (60%)	
Black/African American	3 (15%)	1 (6%)	4 (11%)	
Latino	3 (15%)	1 (6%)	4 (11%)	
Asian/Pacific Islander	5 (25%)	2 (11%)	7 (18%)	.23

Education				

High school/GED	6 (30%)	7 (39%)	13 (34%)	
Associate's/Bachelors Degree	11 (55%)	6 (33%)	17 (44%)	
Graduate School	3 (15%)	5 (28%)	8 (21%)	.37

Marital Status				

Single (never married)	3 (15%)	6 (33%)	9 (24%)	
Married/living together	8 (40%)	9 (50%)	17 (45%)	
Separated/Divorced	9 (45%)	3 (17%)	12 (32%)	.14

Monthly Income				

None	7 (35%)	3 (17%)	10 (26%)	
$500 to $2000	7 (35%)	7 (39%)	14 (37%)	
>$2000	6 (30%)	8 (44%)	14 (37%)	.41
Score (Range: 2-55), Mean (SD)	18.6 (11.1)	18.3 (13.3)	18.4 (12.0)	.95
GAD_7 Score (Range: 0-21), Mean (SD)	9.6 (6.5)	6.2 (5.0)	8.0 (6.0)	.09

Abbreviation: SD, standard deviation.

a P values are based on t-test for continuous variables and chi-square test for categorical variables.

Compared to control group participants, at 12-week follow-up, those in the intervention group more frequently used social media to discuss their pain, prescription opioid use, coping strategies, places to seek help, and alternative therapies for pain. There were no differences between groups in discussions about illegal substances to help address pain (Table 2).

**Table 2:** Social media communications by condition at baseline and follow-up (12 weeks).

Communicated about: n (%)	Baseline				12 weeks			
	Intervention Group (n = 20)	Control Group (n = 18)	Effect size^a^	p-value	Intervention Group (n = 20)	Control Group (n = 18)	Effect size^a^	p-value
Feelings of pain	16 (80%)	11 (61%)	0.21	0.19	19 (95%)	12 (67%)	0.36	0.02
Opioid use	10 (50%)	7 (39%)	0.11	0.49	16 (80%)	5 (28%)	0.52	0.001
Coping strategies	15 (75%)	10 (56%)	0.20	0.21	19 (95%)	11 (61%)	0.42	0.01
Places to seek help	12 (60%)	8 (44%)	0.16	0.34	17 (85%)	7 (39%)	0.48	0.003
Use of illegal drugs for pain	6 (30%)	4 (22%)	0.09	0.59	8 (40%)	3 (17%)	0.25	0.11
Use of alternative therapies	14 (70%)	11 (61%)	0.09	0.56	18 (90%)	10 (56%)	0.39	0.02

a Effect size based on phi coefficient from chi-square tests.

Compared to control group participants, intervention participants showed a baseline-to-follow-up decrease in anxiety, measured by the GAD-7 score (Table 3). The reduction in GAD-7 score from 9.55 to 7.25 roughly translates to a reduction from ‘moderate’ to ‘mild’ anxiety. Participants in both groups showed a baseline to follow-up decrease in opioid misuse, measured by the COMM score. We examined the potential peer-mediated effect on the intervention and found no differences in the COMM and GAD-7 scores across the peer leaders. In addition, we found no differences between groups from baseline to follow-up on BPI or HRQoL items.

**Table 3:** Current Opioid Misuse Measure (COMM) and Generalized Anxiety Disorder (GAD)-7 item Scale by Condition at Baseline and Follow-up.

	Intervention Group (n = 20)			Control Group (n = 18)		
	Baseline	12-weeks	*P* Value^a,b^	Baseline	12 weeks	*P* Value^a,b^
GAD-7, mean (SD)	9.55 (6.48)	7.25 (6.79)	.04	6.22 (4.99)	6.89 (6.33)	.58
COMM, mean (SD)	18.55 (11.05)	13.35 (10.16)	.03	18.28 (13.30)	11.56 (10.73)	.02

Abbreviation: SD, standard deviation.

^a^ P values are based on paired t-test comparing outcomes pre- and post-intervention.

^b^ Groups did not differ on baseline mean scores for opioid misuse or anxiety scores.

## 4 Discussion

A 12-week intervention online support community for chronic pain patients at high risk for opioid misuse resulted in reduced anxiety and more frequent discussions about chronic pain and safe opioid management. Results suggest preliminary support for using online behavior change communities such as HOPE as tools to decrease risk for opioid misuse/abuse and its complications. Participants in both groups experienced a decrease in COMM score, suggesting that merely belonging to an online community designed to discuss chronic pain and reduce opioid misuse risk factors may be able to assist in reducing prescription opioid misuse.

Anxiety is prevalent among chronic pain patients on opioid therapy and may result in co-prescribing of sedatives, a risk factor for opioid overdose [19]. Past studies have demonstrated that the most significant predictor of prescription opioid misuse severity was anxiety [20]. Low-cost behavioral interventions to reduce anxiety among chronic opioid therapy patients may therefore prevent complications of opioid and sedative co-use, including overdose. Effective low-cost behavioral interventions might especially benefit patients within the US, where half (51%) of all opioid prescriptions are given to patients with mood disorders (60 million prescriptions per year) [21].

The ability to use online behavior change and support communities among chronic opioid patients has several immediate implications for managing morbidity and mortality in the United States. First, providers might use peer-led online support communities as a low-cost and scalable referral resource for patients on chronic opioid therapy. Second, health systems and public health officials might integrate online peer support communities as a behavioral tool to supplement standard medical therapies to prevent opioid addiction.

Alternative explanations for the study outcomes include: 1) Baseline GAD-7 scores were higher among the intervention group, making it possible that group differences in anxiety resulted from a control group floor effect in baseline anxiety. However, baseline group differences were not statistically significant. 2) Baseline to follow-up COMM scores diminished in both groups, possibly due to a bias that informing participants they are joining a study on chronic pain and opioid use reduces short-term aberrant medication-taking behaviors. Future research can address this issue by blinding participants to the study purpose. 3) HOPE intervention implementation was slightly different than for previous HOPE studies. For example, peer leader attrition was 63% prior to the start of the study, compared with 10–15% in previous HOPE studies. This difference, resulting primarily from participants’ chronic pain problems may have resulted in a diminished intervention effect compared with other HOPE studies. Future studies can explore whether and how behavior change communities need to be tailored differently for chronic opioid patients than for other patient communities.

This study has limitations. Due to being a pilot study, the analysis was limited by sample size. However, the present study has a larger sample size than 4 out of the 5 (80%) other published opioid interventions at the time of this submission, demonstrating the need for larger studies. Second, the study lacks an offline comparison group. In previous HOPE studies, control groups were sometimes composed of an online control group (as in this study) and other times of an offline control group. Future research on this topic could include an offline control group to better understand whether the online communities, compared to offline resources, might be more beneficial for behavioral interventions among chronic opioid patients. Only a small fraction of the screened patients were enrolled in the study and therefore results may not be generalizable to all patients with chronic pain. Finally, the study included a post-intervention follow-up of 3 months, limiting our knowledge of whether these effects might persist long term.

Overall, results from this pilot intervention suggest that online communities are an emerging tool that should be evaluated for their potential to reduce opioid misuse risk factors, including anxiety. The 12-week intervention resulted in significant reductions in anxiety and increased discussions about the challenges and treatments for chronic pain and opioid use among non-cancer pain patients who had been prescribed opioids. In our pilot study, we observed a significant reduction in anxiety in the intervention group, as indicated by a reduction in GAD-7 score from 9.55 to 7.25. This significant reduction is meaningful in that it translates to reduction from ‘moderate’ to ‘mid’ anxiety. This finding is promising, but given that our study was a small pilot study, our findings underscore the need for future research on this topic with a larger patient sample and longer follow-up time points.

## Declarations

### Author contribution statement

Sean D. Young, Lillian Gelberg, Keith Heinzerling: Conceived and designed the experiments; Performed the experiments; Analyzed and interpreted the data; Contributed reagents, materials, analysis tools or data; Wrote the paper.

Sung-Jae Lee: Analyzed and interpreted the data; Contributed reagents, materials, analysis tools or data; Wrote the paper.

Hendry Perez, Navkiran Gill: Performed the experiments; Contributed reagents, materials, analysis tools or data.

### Funding statement

The National Institute on Drug Abuse (1R21DA039458), National Institute of Allergy and Infectious Diseases (5R01AI132030), National Institute of Mental Health (5R01MH106415), and National Center for Complementary and Integrative Health (1R61AT010606). The funders played no role in the study planning, analysis, or outcomes.

### Competing interest statement

The authors declare no conflict of interest.

### Additional information

No additional information is available for this paper.
